# Hemophagocytic Lymphohistiocytosis Secondary to Disseminated Bacillus Calmette–Guérin (BCG): A Steroid‐Sparing Strategy in a Patient Awaiting Cardiac Surgery—A Case Report

**DOI:** 10.1155/crh/5761690

**Published:** 2026-02-09

**Authors:** Kaitlin Quinlan, Tyler MacDonald, Terence C. Wuerz, Ryan Zarychanski

**Affiliations:** ^1^ Department of Internal Medicine, Max Rady College of Medicine, University of Manitoba, Winnipeg, Manitoba, Canada, umanitoba.ca; ^2^ Department of Medical Oncology and Haematology, CancerCare Manitoba, Winnipeg, Manitoba, Canada, cancercare.mb.ca; ^3^ Department of Community Health Sciences, Max Rady College of Medicine, University of Manitoba, Winnipeg, Manitoba, Canada, umanitoba.ca

**Keywords:** anakinra, Bacillus Calmette–Guérin, case report, hemophagocytic lymphohistiocytosis

## Abstract

We report a case of hemophagocytic lymphohistiocytosis (HLH) secondary to disseminated Bacillus Calmette–Guérin (BCG) infection in an 80‐year‐old man treated with intravesical BCG for non–muscle‐invasive bladder cancer. The patient presented with several weeks of constitutional symptoms including night sweats, fatigue, weight loss, confusion, and pancytopenia. Laboratory studies revealed profound inflammation, coagulopathy, and hepatocellular injury. HLH was suspected clinically and supported by a high H‐score. Extensive infectious and autoimmune workup was negative. *Mycobacterium bovis* was subsequently isolated from urine and bone marrow cultures, which confirmed a diagnosis of disseminated BCG infection.

The patient was treated with a combination of antimycobacterial therapy (isoniazid, rifampin, and ethambutol), intravenous immunoglobulin (IVIG), corticosteroids, and anakinra. Anakinra, an interleukin‐1 receptor antagonist, was used both to minimize high‐dose steroids, given the protracted inflammatory response anticipated with *Mycobacterium* infection, and to prevent steroid‐related complications related to cardiac surgery. This therapeutic strategy was associated with improvement of cytopenias, normalization of inflammatory markers, and gradual clinical recovery. This case highlights the rare but serious complication of HLH triggered by disseminated BCG and suggests that targeted immunomodulation with anakinra may be a useful adjunct in selected, complex presentations of infection‐associated HLH.

**Key Points**
1. Disseminated BCG is an uncommon but serious complication of intravesical therapy, associated with a high mortality rate.
2. Hemophagocytic lymphohistiocytosis (HLH), a syndrome associated with hyperinflammation and organ dysfunction due to immune system activation in conjunction with inadequate immune downregulation, can be triggered by disseminated BCG infection.
3. If HLH with organ dysfunction secondary to disseminated BCG infection is suspected, prompt initiation of empirical antituberculous therapy in addition to treatment of HLH is recommended.
4. Anakinra, an interleukin‐1 receptor antagonist, is a useful adjunct to corticosteroids, especially in cases where prolonged administration of steroids is expected or contraindicated.

## 1. Case Presentation

An 80‐year‐old man with localized urothelial carcinoma previously treated with transurethral resection and intravesical Bacillus Calmette–Guérin (BCG) therapy presented with several weeks of constitutional symptoms including drenching night sweats, profound fatigue, and an unintentional 4.5‐kg loss. He had completed his sixth intravesical BCG treatment 2 weeks prior but had poorly tolerated the last two cycles. On examination, he was found to be febrile and confused and had diffuse abdominal pain. He had no signs or symptoms of pulmonary, urinary, or central nervous system infection. His past medical history was significant for a remote history of prostate cancer, which was treated with brachytherapy.

Initial laboratory evaluation revealed pancytopenia with a white blood cell count of 2.5 × 10^9^/L (normal: 4.0–11.0 × 10^9^/L), a hemoglobin level of 105 g/L (normal: 120–160 g/L), and a platelet count of 114 × 10^9^/L (normal: 150–400 × 10^9^/L). Inflammatory markers were elevated, including C‐reactive protein of 14 mg/L (normal: < 5 mg/L), ferritin of 2756 μg/L (normal: 30–300 μg/L), and D‐dimer of 1195 ng/mL (normal: < 500 ng/mL). Coagulopathy was evident, with a fibrinogen level of 1.2 g/L (normal: 2.0–4.0 g/L). Evidence of hepatocellular injury and cholestasis was present: aspartate aminotransferase 202 U/L (normal: < 40 U/L), alanine aminotransferase 206 U/L (normal: < 45 U/L), alkaline phosphatase 358 U/L (normal: 30–120 U/L), gamma‐glutamyl transferase 333 U/L (normal: < 40 U/L), lactate dehydrogenase 614 U/L (normal: 120–250 U/L), and total bilirubin 21 μmol/L (normal: < 20 μmol/L). Computed tomography showed splenomegaly (15 cm), chronic bladder wall thickening, and acute cholecystitis. Blood and urine cultures were negative. He underwent an uncomplicated laparoscopic cholecystectomy and was then admitted for further workup of his pancytopenia. The tissue specimen from his cholecystectomy revealed hyperplastic mucosal changes without evidence of inflammation.

Hemophagocytic lymphohistiocytosis (HLH) was suspected with an H‐score of 253, corresponding to a ∼99% probability of HLH [1]. Extensive workup excluded viral triggers: cytomegalovirus (CMV) IgM, Epstein–Barr virus (EBV) PCR, human immunodeficiency virus (HIV) antigen/antibody, and hepatitis B and C serologies. Autoimmune markers, including antinuclear antibody, antineutrophil cytoplasmic antibody, rheumatoid factor, antimitochondrial antibody, smooth muscle antibody, and anti–liver–kidney microsomal antibody, were negative. Bone marrow biopsy demonstrated trilineage hematopoiesis with hemophagocytosis, without dysplasia or malignancy (see​ Figure 1).

FIGURE 1Clinical photograph showing the patient’s pathology findings. (a) Wright–Giemsa stain of bone marrow aspirate showing a large pale‐staining macrophage in the lower right with abundant vacuolated cytoplasm and a central nucleus, demonstrating hemophagocytosis of hematopoietic cells. (b) Wright–Giemsa stain of bone marrow aspirate showing numerous activated macrophages with engulfed hematopoietic cells, consistent with marked hemophagocytosis. (c) AFB stain (at low power) showing blue‐stained marrow background with scattered red–pink rod‐shaped acid‐fast bacilli consistent with mycobacterial organisms. (d): H&E staining of the core biopsy shows a caseating granuloma fragment, with central necrosis surrounded by activated macrophages, suggestive of a mycobacterial infection.

**Figure:**
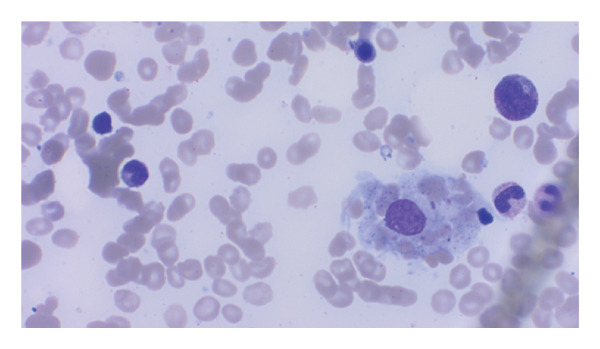
(a)

**Figure:**
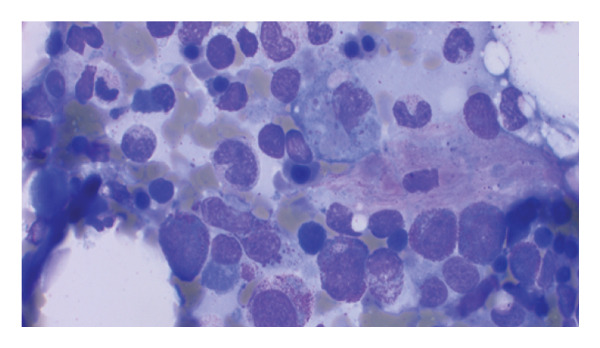
(b)

**Figure:**
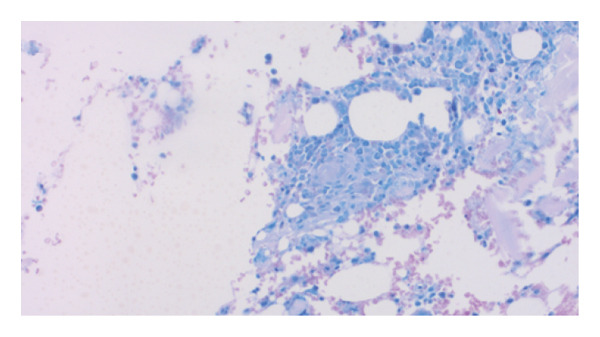
(c)

**Figure:**
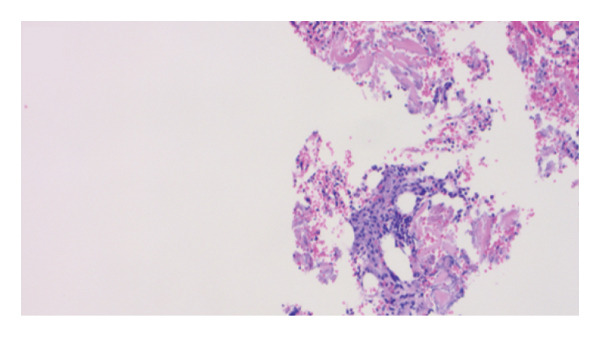
(d)

Given the history of BCG therapy, disseminated *Mycobacterium bovis* infection was suspected. Treatment included oral isoniazid, rifampin, and ethambutol, intravenous immunoglobulin (IVIG) 400 mg/kg for five doses, and prednisone 70 mg daily. Incidentally, later in the admission, imaging revealed an 8‐cm aortic root aneurysm, with moderate aortic regurgitation. Magnetic resonance imaging showed no evidence of aortitis. Cardiac surgery was consulted and recommended urgent aneurysm repair. To accommodate the urgent procedure, steroids were tapered over 11 days, and anakinra, an interleukin‐1 receptor antagonist, was initiated at 100 mg subcutaneously twice daily and later increased to 200 mg three times daily, achieving HLH control while minimizing perioperative immunosuppressive risk.

**FIGURE 2:**
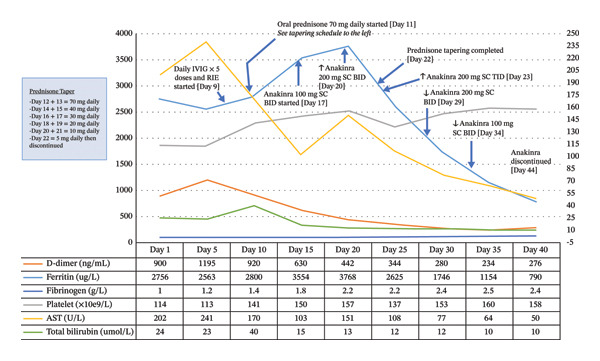
Timeline of hospital laboratory values over 40 days showing improvement in HLH‐associated biomarkers with initiation of immunomodulatory therapy. D‐dimer, ferritin, fibrinogen, platelets, AST, and bilirubin are plotted against time, with treatment interventions (IVIG, RIE, prednisone, and anakinra) indicated by arrows. IVIG, intravenous immunoglobulin; SC, subcutaneously; BID, twice daily; TID, three times daily; RIE, rifampin, isoniazid, and ethambutol; AST, aspartate aminotransferase.

Over the subsequent weeks, the patient’s pancytopenia resolved, and liver transaminases as well as inflammatory markers returned to baseline levels. See Figure 2 for a summary infographic of laboratory timeline and treatment history. Subsequently, *M. bovis* was isolated from both urine and bone marrow cultures obtained prior to the initiation of antimycobacterial therapy, confirming disseminated BCG infection associated with HLH. Anakinra was continued at 100 mg subcutaneously twice daily while the patient awaited cardiac surgery.

## 2. Discussion

### 2.1. HLH

HLH is a rare, life‐threatening hyperinflammatory syndrome characterized by uncontrolled activation of the immune system, leading to severe cytokine release and multiorgan dysfunction, which is associated with high mortality [2]. Unlike in young children, where HLH is often the result of inherited mutations, most cases in adults are triggered by infection, malignancy, or autoimmune disease [2]. While no diagnostic gold standard definition exists, a consensus‐based diagnosis of HLH can be made using the HLH‐2004 criteria (a set of eight clinical, laboratory, and histopathologic features, of which at least five must be present) or the H‐score (a weighted scoring system incorporating clinical, laboratory, and cytologic variables to estimate the probability of secondary HLH) [3]. However, the H‐score’s performance is limited by the absence of a gold standard test and clinical overlap with other inflammatory conditions and confounding illnesses.

In adults, HLH may be triggered by a wide range of conditions. Primary (familial) HLH is rare but possible, often linked to mutations such as *PRF1*, *UNC13D*, and *STX11* [2]. Infections are common precipitants, particularly due to viruses (e.g., EBV, CMV, herpes simplex virus, HIV, hepatitis viruses, and parvovirus B19), but also bacteria (e.g., *Mycobacterium tuberculosis*, *Rickettsia*, *Escherichia coli*, *Staphylococcus* spp*.*, and *Brucella*), fungi, or parasites (e.g., *Histoplasma*, *Leishmania*, *Toxoplasma*, and *Plasmodium*) [2]. Malignancy‐associated HLH occurs most often with hematologic cancers, especially T‐cell or NK‐cell lymphomas, but may also be seen with leukemia, Hodgkin’s lymphoma, Castleman’s disease, and rarely solid tumors [2]. Autoimmune‐ or rheumatic disease–associated HLH is linked to conditions such as SLE, adult‐onset Still’s disease, rheumatoid arthritis, and vasculitis [2]. Drug‐related or iatrogenic HLH can follow biologic therapies (e.g., anti‐TNF agents and IL‐6 inhibitors), chemotherapy, transplantation, immune checkpoint inhibitors, or hemodialysis [2]. Around 25% of cases are idiopathic, prompting evaluation for occult malignancy, infection, or genetic predisposition. Other less common triggers include pregnancy, diabetes, chronic liver disease, and postoperative states [2].

### 2.2. Disseminated BCG and HLH

Intravesical administration of BCG (a live, attenuated strain of *M. bovis*) is part of the standard initial treatment for superficial non–muscle‐invasive urothelial carcinoma. While adverse effects are relatively common, they are typically localized to the bladder (BCG cystitis) and self‐limited [4]. Severe systemic complications are rare and occur in fewer than 5% of cases [5]. In a large cohort of 6753 patients, the incidence of systemic BCG‐related illness was reported in 1%, with a median onset of approximately 170 days following the last instillation [6]. Delayed‐onset cases may arise from reactivation of latent *M. bovis* infection or manifest as a late‐onset hypersensitivity reaction. Both can be challenging to diagnose due to the prolonged interval typically present between inflammatory symptoms and prior BCG exposure. Table A1 in the appendix lists the complications associated with intravesical BCG, as well as their relative frequencies. Risk factors for systemic complications of intravesical BCG are provided in Box 1. International urologic guidelines recommend delaying intravesical BCG instillation in the presence of gross hematuria, traumatic catheterization, active urinary tract infection, and for at least 2 weeks after TURBT to minimize the risk of systemic BCG infection [7].

Systemic symptoms can arise not only from dissemination of *M. bovis* but also from an exaggerated host‐immune response to the organism. The range of potential complications of intravesical BCG is listed in Table A1 of the appendix. Diagnosing systemic complications of BCG instillation can be challenging; therefore, multiple specimens should be collected, including blood and bone marrow, as well as sputum and urine cultures if those sites are clinically involved. Limited published reports document HLH following intravesical BCG. As no randomized trials specifically guide the management of disseminated BCG infection, clinical judgment remains central to therapeutic decision‐making.

Box 1. Risk factors for disseminated BCG infection.

• Recent TURBT/traumatic catheterization: ↑ systemic BCG absorption
• Early postsurgery instillation: incomplete mucosal healing
• Active UTI or hematuria: facilitates bloodstream entry
• Immunosuppression: steroids, chemotherapy, or immune compromise
• Older age: higher risk of systemic complications

In 2023, Güven et al. reported a 65‐year‐old man with low‐grade papillary urothelial carcinoma receiving intravesical BCG who developed autoimmune hemolytic anemia (hemoglobin 109 g/L), pancytopenia, hyperferritinemia, and abnormal liver function tests [15]. Although triglyceride levels were not provided, the partial calculated H‐score was 156, indicating a moderate probability of HLH. Empiric treatment with isoniazid, rifampicin, and ethambutol was initiated, and bone marrow biopsy subsequently revealed multinucleated giant cells with abortive granuloma formation. Glucocorticoids were withheld due to stable hemoglobin and platelet counts and the absence of organ dysfunction. The patient demonstrated progressive clinical and biochemical improvement on antimycobacterial therapy alone over six weeks and ultimately returned to baseline health [15].

Although antimycobacterial therapy alone may be sufficient in milder cases, individuals presenting with severe systemic inflammation with end‐organ dysfunction may benefit from the addition of immunosuppressive therapy [8, 15]. In 2002, Schleinitz first described a 49‐year‐old male who developed severe HLH one month after the last BCG instillation. Treatment included high‐dose prednisone (1 mg/kg daily), a 4‐day course of IVIG 400 mg/kg per day, and a four‐drug antitubercular regimen (isoniazid, rifampin, ethambutol, and pyrazinamide). Vinblastine was also administered weekly for immunosuppression [4]. Subsequently, three additional severe cases were reported, with symptom onset ranging from a few hours to 15 days after intravesical BCG instillation, all of which responded to corticosteroids, IVIG, and antituberculous therapy [6, 9, 10]. Of note, pyrazinamide is not recommended in disseminated BCG infection. As an attenuated derivative of *M. bovis*, BCG is intrinsically resistant to pyrazinamide [15].

The most severe outcome was noted in a case published by Stojanovic et al., in which a 55‐year‐old man developed fulminant multiorgan failure 1 week after his second BCG instillation cycle [11]. On admission, he received rifampin in combination with amikacin and ceftriaxone. On Day 2, IVIG and methylprednisolone were added due to clinical deterioration [11]. Dual antibiotics and rifampin were subsequently discontinued, and therapy was escalated to include meropenem, linezolid, and metronidazole; however, the patient died on the sixth day of hospitalization [11].

Although detailed corticosteroid tapering regimens were not specified in any of the published reports, the treatment of HLH often requires prolonged immunosuppression. Given the expected gradual clinical response to antituberculous drugs combined with the need for expedited cardiac surgery, a steroid‐sparing approach was chosen for our patient. Prednisone was tapered over 11 days, while anakinra was uptitrated to prevent recrudescence of inflammation.

HLH is driven by hyperactivation of immune cells, with interferon‐gamma signaling playing a central role in the inflammatory cascade. Anakinra, an interleukin‐1 receptor antagonist, targets this pathway, offering a more specific approach than corticosteroids [12]. There are currently no published randomized controlled trials evaluating anakinra specifically for HLH; however, retrospective observational studies demonstrate rapid clinical responses and encouraging rates of remission with anakinra, often avoiding the need for more toxic agents such as etoposide [12, 13].

Published studies and practice guidelines support the use of anakinra in both primary and secondary HLH, especially when minimizing infection and perioperative risks [14]. Compared with corticosteroids, anakinra is associated with a lower risk of surgical complications [14]. In our patient, the need for urgent cardiac surgery while concomitantly managing active HLH made a targeted agent ideal. Anakinra’s short half‐life and selective immunomodulation allowed treatment continuation up to surgery with minimal impact on wound healing or infection [13]. Corticosteroids, by contrast, impair multiple phases of healing, increase the risk of sepsis and hemodynamic instability, and require careful perioperative tapering to avoid adrenal insufficiency.

In conclusion, HLH secondary to disseminated BCG infection is a rare and potentially life‐threatening complication of intravesical BCG. This single‐patient report with limited follow‐up cannot establish causality between specific therapies and clinical improvement. However, the case highlights a practical, individualized management approach in which targeted immunomodulation with anakinra was used alongside antimycobacterial therapy, corticosteroids, and IVIG. In this context, anakinra appeared to contribute to disease control while allowing a more rapid steroid taper in a patient requiring urgent cardiac surgery. These observations support the need for further study of cytokine‐directed therapies as adjuncts in complex or high‐risk presentations of infection‐associated HLH.

## Funding

No funding was obtained for this case report.

## Disclosure

This case report was structured and written in accordance with the CARE checklist to ensure comprehensive and transparent reporting of patient information, clinical findings, interventions, and outcomes. The case has been anonymized in accordance with ICMJE guidelines to avoid patient identification.

## Consent

No written consent has been obtained from the patients as there are no patient identifiable data included in this case report/series.

## Conflicts of Interest

The authors declare no conflicts of interest

## Data Availability

The data that support the findings of this study are available upon request from the corresponding author. The data are not publicly available due to privacy or ethical restrictions.

## Appendix

**TABLE A1:** Complications of intravesical Bacillus Calmette–Guérin

Type of complication	Specific complications	Frequency/notes	Management
Local complications	‐Cystitis (dysuria, frequency, urgency)‐Hematuria	Very common (up to 90%)	Usually self‐limited; symptomatic treatment with NSAIDs, anticholinergics
	‐Bladder contracture, fibrosis	Rare, serious	May require surgery (e.g., cystectomy)
	‐BCG granulomas in the bladder	Rare	Usually resolves with cessation of therapy

Regional complications	‐Epididymo‐orchitis	Rare, 0.4%–5%	Antimycobacterial therapy and symptomatic care
	‐Prostatitis	Rare	Antimycobacterial treatment; often difficult to distinguish from cancer

Systemic complications	‐Fever and flu‐like symptoms	Common, mild	Symptomatic treatment, temporary BCG interruption if severe
	‐Disseminated BCG infection	Very rare (< 1%)	Prolonged antimycobacterial therapy; hospitalization often needed
	‐Pneumonitis, hepatitis, sepsis, bone marrow suppression	Rare	Aggressive antimycobacterial and supportive care

Immunologic/hypersensitivity	‐Reactive arthritis	Rare	NSAIDs, corticosteroids
	‐Vasculitis	Very rare	Corticosteroids, immunosuppressive agents

Other organ complications	‐Granulomatous hepatitis	Rare	Antimycobacterial therapy
	‐Osteomyelitis	Very rare	Long‐term antimycobacterial therapy
	Renal granulomas/interstitial nephritis	Very rare	Antimycobacterial therapy and supportive care

Complications related to administration	‐Sepsis due to traumatic catheterization or overdose	Rare	Prevention: careful catheterization technique; antimycobacterial therapy if occurs
	‐Allergic reactions to BCG material	Uncommon	Supportive care; corticosteroids if severe

*Note:* Table A1 Reference: Macleod, L.C., Ngo, T.C., & Gonzalgo, M.L. (2014). Complications of intravesical Bacillus Calmette–Guérin. Canadian Urological Association Journal, 8(7–8), e540–e544. https://doi.org/10.5489/cuaj.1411.
